# Robot-assisted radical nephrectomy in comparison with open and laparoscopic approaches: a Japanese single-institution retrospective study

**DOI:** 10.1007/s11701-025-02898-x

**Published:** 2025-11-03

**Authors:** Tatsuya Umemoto, Airi Ichige, Naoko Matsumoto, Daichi Nishikawa, Kumpei Takahashi, Soichiro Yuzuriha, Tatsuya Otaki, Nobuyuki Nakajima, Yoshiaki Kawamura, Sunao Shoji

**Affiliations:** 1 Department of Urology, Tokai University School of Medicine, Kanagawa, Japan; 2 Department of Urology, Tokai University School of Medicine, 143 Shimokasuya, Isehara, 259-1193 Kanagawa Japan

**Keywords:** Robot-assisted surgery, Radical nephrectomy, Renal cell carcinoma, Perioperative outcomes

## Abstract

**Objective:**

To appraise perioperative metrics and oncologic results for robot-assisted radical nephrectomy (RARN) in comparison with open radical nephrectomy (ORN) and laparoscopic radical nephrectomy (LRN) based on real-world experience in Japan.

**Methods:**

We conducted a retrospective appraisal of 165 radical nephrectomy patients managed at a single center over 2015–2025: 36 RARN, 33 ORN, 96 LRN. Patient backgrounds, perioperative outcomes, and disease-free survival (DFS) rates were compared. Propensity score matching (PSM) was performed based on tumor size, ≥pT2 status, and presence of venous tumor thrombi.

**Results:**

Compared with ORN, RARN was associated with significantly reduced operative times (median: 180 vs. 235 min, *p* = 0.018), blood loss (58 vs. 1,187 g, *p* < 0.001), and hospital stays (6.5 vs. 10 days, *p* < 0.001). In contrast, LRN demonstrated superior perioperative outcomes compared with RARN in terms of operative times, blood loss, and length of hospital stay. Even after PSM, LRN maintained shorter hospital stays than RARN. Notably, although patients undergoing RARN exhibited a higher prevalence of complex renal tumors, including ≥ pT2 disease and venous tumor thrombi, they still showed favorable outcomes compared with ORN, highlighting its feasibility in high-risk surgical cases. Two-year DFS rates did not differ significantly between groups after adjustment.

**Conclusion:**

RARN provides reliable safety and clinical effectiveness in treating advanced or technically challenging renal tumors. However, given its resource intensity and lengthier procedures, LRN is still a suitable approach in low-risk settings. The surgical approach should be selected based on tumor complexity and resource considerations.

**Supplementary Information:**

The online version contains supplementary material available at 10.1007/s11701-025-02898-x.

## Introduction

Renal cell carcinoma (RCC) accounts for about 2–3% of adult cancers, and radical nephrectomy is typically selected for sizable or centrally located non-metastatic tumors [1]– [2].Historically, open radical nephrectomy (ORN) was considered the reference procedure, while adoption of laparoscopic radical nephrectomy (LRN) expanded with the growth of minimally invasive surgery due to reduced invasiveness and faster convalescence [3].

In recent years, robot-assisted radical nephrectomy (RARN) has become increasingly popular worldwide because it offers improved surgeon ergonomics, superior instrument maneuverability, and 3D visualization [4]. Reports from Western cohorts indicate that RARN provides oncological results similar to ORN and LRN, while often yielding advantages such as reduced blood loss and shorter hospitalization [5]. In Japan, RARN was approved for use in 2022, and its use has increased rapidly. However, real-world comparative data on RARN and conventional surgical approaches remain scarce, especially in the context of single-institution retrospective analyses [6].

An additional challenge in assessing the benefits of RARN is potential case selection bias. In clinical practice, RARN is often chosen for anatomically complex or locally advanced tumors, which may influence the interpretation of perioperative outcomes [7]. Therefore, directly comparing these surgical techniques without accounting for tumor complexity or technical difficulty may lead to biased assessments of their safety and effectiveness.

To address these concerns, the current study sought to evaluate and benchmark perioperative and oncologic outcomes across RARN, ORN, and LRN, using real-world data from a Japanese tertiary referral center. Furthermore, we performed propensity score matching (PSM) to control for baseline differences in tumor complexity and patient characteristics, thereby enabling a more robust and clinically relevant comparison among the three surgical modalities.

## Materials and methods

### Study design and patient selection

This retrospective, single-institutional study was conducted at a tertiary referral center in Japan. We reviewed the medical records of all patients who underwent radical nephrectomies for RCC between January 2015 and May 2025. The analytic cohort comprised 165 patients; 33 underwent ORNs (2015–2025), 96 underwent LRNs (2020–2025), and 36 underwent RARNs (2023–2025). Patients who underwent a partial nephrectomy or had incomplete clinical data were excluded. Cytoreductive nephrectomy (CN) cases were included if performed for metastatic RCC; however, RARN and LRN cases included only deferred CN cases, whereas the ORN group included both upfront and deferred CN procedures.

### Surgical techniques

RARN was undertaken with the da Vinci Xi via a transperitoneal route. Patients were placed laterally, and four robotic trocars plus an assistant trocar were used (an extra trocar was added for right-sided procedures). Vascular control of the renal vessels was achieved using Hem-o-lok^®^ clips rather than energy devices.

In the context of venous tumor thrombus, the IVC was exposed and secured prior to thrombectomy, following established robotic vascular control techniques reported for complex cases. The surgical technique used in our institution was similar to that described by Petros et al. and Gu et al., which demonstrated the feasibility and safety of robot-assisted radical nephrectomy, including cases with IVC tumor thrombi [7, 8]. LRN was undertaken via either the transperitoneal or the retroperitoneal route; five of the 96 LRN cases were conducted retroperitoneally. ORN was performed using a standard open technique, and a retroperitoneal approach was used in only one case. Lymph node dissection was selectively performed in patients with radiologically apparent lymph node metastases on preoperative computed tomography (CT).

In cases of venous tumor thrombi, surgical management varied according to the approach. LRN was not performed for disease with *caval* tumor thrombus extending past the renal vein; only patients with thrombi confined to the renal vein, amenable to standard vascular control, were included in the LRN group. In contrast, RARN was actively performed in selected cases of IVC tumor thrombi up to Mayo level II, utilizing standard robotic techniques for vascular control and thrombectomy.

### Clinical variables and outcomes

Data were collected on patient demographics, tumor characteristics (including tumor size, laterality, and clinical T stage), and perioperative outcomes (operative time, estimated blood loss, length of hospital stay, and intraoperative/postoperative complications). Complications were classified according to the Clavien–Dindo grading system.

We included patients with both non-metastatic and metastatic RCC who underwent CNs. Therefore, progression-free survival (PFS) was used as the primary oncologic endpoint, defined as the time from surgery to clinical or radiographic progression. Follow-up evaluations were performed using cross-sectional imaging according to the institutional protocol.

### Propensity score matching

To minimize selection bias, the primary RARN-LRN analysis used propensity score matching (PSM) across the entire study period. Propensity scores were estimated using a logistic model that included tumor size (continuous), pathological T stage (≥ pT2), and the presence of venous tumor thrombi. We performed 1:1 nearest-neighbor matching without replacement with a caliper of 0.20 standard deviations on the logit-transformed propensity score. Covariate balances were assessed using standardized mean differences (SMDs) with a target of < 0.10; baseline p-values were not used. All outcome analyses were conducted on the matched cohorts.

In complementary sensitivity analyses, we (i) restricted the cohort to 2023–2025 to address potential temporal and learning-curve biases, (ii) excluded the first five RARN cases, and (iii) added metastatic status (M0/M1) to the propensity model. The results of these analyses are presented in the Supplemental Materials.

### Statistical analyses

Statistical analyses were carried out with EZR (version 1.68; RcmdrPlugin.EZR, Yoshinobu Kanda, Saitama Medical Center, Jichi Medical University, Saitama, Japan), which serves as a graphical user interface to R (version 4.2.3; R Foundation for Statistical Computing). Continuous variables were compared using the Mann–Whitney U test, whereas categorical variables were assessed with the chi-square (χ²) test or Fisher’s exact test when appropriate. Time-to-event outcomes were summarized by the Kaplan–Meier method, and between-group differences were evaluated using the log-rank test. Unless otherwise specified, two-sided testing was adopted and statistical significance was defined as *p* < 0.05 (two-sided).

## Results

### Patient characteristics

The baseline characteristics of the patients who underwent RARN and LRN are shown in Table 1. No significant differences were observed regarding age, tumor laterality, or body mass index between the two groups. However, patients in the RARN group had significantly larger tumors, higher rates of ≥ pT2 disease, a greater incidence of venous tumor thrombi, and more metastatic disease than those in the LRN group.

**Table 1:** Clinical characteristics of patients treated with robot-assisted versus laparoscopic radical nephrectomy prior to propensity score adjustment

Variables	RARN (*n* = 35)	LRN (*n* = 96)	*p*-value
Age (years), median (IQR)	70 (65–75)	71 (56–76)	0.747
Laterality (Left/Right)	17/18	51/45	0.695
BMI, median (IQR)	23 (19.5–24.5)	23 (21.0–26.0)	0.300
Tumor diameter (mm), median (IQR)	65.0 (45.0–87.5)	43.5 (35.0–57.8)	0.002
≥ pT2, n (%)	23	32	0.001
History of abdominal surgery, n	6	12	0.568
Venous tumor thrombus, n	8	2	< 0.001
Neoadjuvant therapy, n	15	10	< 0.001
Metastatic disease, n	10	4	< 0.001

Values are reported as medians with interquartile ranges, or as counts with corresponding percentages

Table 2 shows the comparison between the RARN and ORN groups. The ORN group had significantly larger tumors and a higher proportion of ≥ pT2 disease.

**Table 2:** Clinical features of patients who underwent robot-assisted versus open radical nephrectomy prior to propensity score adjustment

Variables	RARN (*n* = 35)	ORN (*n* = 33)	*p*-value
Age (years), median (IQR)	70 (65–75)	71 (53–75)	0.277
Laterality (Left / Right)	17 / 18	13 / 20	0.474
BMI, median (IQR)	23 (19.5–24.5)	22 (20.8–25.0)	0.940
Tumor diameter (mm), median (IQR)	65.0 (45.0–87.5)	100.0 (70.0–115.0)	0.002
≥ pT2, n (%)	23	31	0.006
History of abdominal surgery, n	6	8	0.555
Venous tumor thrombus, n	8	15	0.073
Neoadjuvant therapy, n	15	8	0.129
Metastatic disease, n	10	8	0.786

Values are summarized as medians with interquartile ranges or as counts with percentages

### Post-propensity score matching characteristics

Following PSM based on tumor size, ≥pT2 stage, and presence of venous tumor thrombi, 24 matched pairs were identified in the RARN vs. LRN comparison (Table 3). The baseline characteristics were largely balanced, although the RARN group had a significantly higher number of patients with metastatic disease (*p* = 0.048).

**Table 3:** Patient characteristics for robot-assisted versus laparoscopic radical nephrectomy following propensity score matching

Variables	RARN (*n* = 24)	LRN (*n* = 24)	*p*-value
Age (years), median (IQR)	70.5 (65.0–75.0)	75.0 (70.8–80.1)	0.189
Laterality (Left/Right)	11/13	10/14	0.564
BMI, median (IQR)	22.5 (19.8–24.5)	23.0 (22.0–27.0)	0.117
Tumor diameter (mm), median (IQR)	50.0 (31.5–65.5)	47.0 (31.8–70.0)	0.893
≥ pT2, n (%)	13	14	1.0
History of abdominal surgery, n	3	3	1.0
Venous tumor thrombus, n	1	1	1.0
Neoadjuvant therapy, n	7	5	0.74
Metastatic disease, n	7	1	0.048

Data are displayed as medians with interquartile ranges, or as numbers with percentages. Propensity score matching was conducted considering tumor size, pT2 or higher status, and venous tumor thrombus

In the RARN vs. ORN comparison (Table 4), 18 matched pairs were generated. Although the ORN group included significantly more patients who received neoadjuvant therapy (*p* = 0.0176), we observed no statistically significant divergence in metastatic case counts between the groups (Table 4).

**Table 4:** Patient features of robot-assisted versus open radical nephrectomy after propensity score adjustment

Variables	RARN (*n* = 18)	ORN (*n* = 18)	*p*-value
Age (years), median (IQR)	68 (61.8–75.0)	69 (53.8–72.8)	0.303
Laterality (Left/Right)	10/8	8/10	0.74
BMI, median (IQR)	21.5 (19–24)	22.0 (21–25)	0.561
Tumor diameter (mm), median (IQR)	85 (68.5–110.0)	85 (66.3–103.8)	0.728
≥ pT2, n (%)	15	16	1.0
History of abdominal surgery, n	3	3	1.0
Venous tumor thrombus, n	8	8	1.0
Neoadjuvant therapy, n	4	12	0.0176
Metastatic disease, n	6	6	1.0

Data are displayed as medians with interquartile ranges, or as numbers with percentages. Propensity score matching used tumor size, stage ≥ pT2, and the presence of venous tumor thrombus as covariates

### Perioperative outcomes

#### RARN vs. LRN

Unmatched cohort: In the unmatched cohort, the LRN group had significantly shorter operative times, lower estimated blood loss (EBL), and shorter postoperative hospital stays than the RARN group. Lymph node dissection was performed more frequently in the RARN group (Supplemental Table 1).

PSM cohort (primary, entire period): After PSM across the entire study period (Table 5), postoperative hospital stay remained significantly shorter with LRN, whereas operative time and EBL were comparable between groups.

**Table 5:** Perioperative results in patients treated with robot-assisted versus laparoscopic radical nephrectomy after propensity score adjustment

Variables	RARN (*n* = 24)	LRN (*n* = 24)	*p*-value
Operative time (min), median (IQR)	172.0 (132.8–203.8)	156.5 (126.5–179.3)	0.164
Console time (min), median (IQR)	96 (75.8–134)	–	–
Estimated blood loss (mL), median (IQR)	54.0 (3.5–142.5)	7.5 (3.0–34.3)	0.166
Postoperative hospital stay (days), median (IQR)	6.0 (4–9.3)	5.0 (4–5)	0.022
Complications (≥ grade 3), n	2	2	1.0
Postoperative recurrence/metastasis, n	6	6	1.0

Data are displayed as medians with interquartile ranges, or as numbers with percentages

Propensity score matching used tumor size, stage ≥ pT2, and the presence of venous tumor thrombus as covariates

Sensitivity analysis (time-restricted [2023–2025]): Adequate covariate balance was achieved in this matched cohort (all SMDs < 0.10; Supplemental Table 3a and b). When restricting the analysis to 2023–2025, the difference in hospital stays attenuated and was no longer significant. Operative times showed no statistically significant difference but were numerically shorter with RARN, and EBL remained comparable (Supplemental Table 3c).

Sensitivity analysis (excluding the overall first five RARN cases): Adequate covariate balance was achieved (all SMDs < 0.10; Supplemental Table 4a, b). Excluding the overall first five RARN cases yielded a similar pattern: the between-group difference in hospital stay disappeared, operative time was again numerically shorter with RARN without statistical significance, and EBL was comparable (Supplemental Table 4c).

#### RARN vs. ORN

In the unmatched RARN vs. ORN comparison, RARN demonstrated superior perioperative outcomes, with a shorter operative time, reduced EBL, shorter length of hospital stay, and fewer cases of recurrence/metastases (Supplementary Table 1b). Following PSM (Table 6), the RARN group had significantly lower EBL. Although not statistically significant, the RARN group showed trends toward shorter operative times, reduced length of hospital stay, and fewer Clavien–Dindo grade ≥ 3 complications.

**Table 6:** Perioperative results for robot-assisted versus open radical nephrectomy following propensity score matching

Variables	RARN (*n* = 18)	ORN (*n* = 18)	*p*-value
Operative time (min), median (IQR)	186 (161.0–282.3)	248 (178.8–386.8)	0.308
Console time (min), median (IQR)	118.5 (90–214.3)	–	–
Estimated blood loss (mL), median (IQR)	111.5 (25.3–154.5)	1061.0 (389.5–2659.5)	< 0.001
Postoperative hospital stay (days), median (IQR)	8.5 (6–10.8)	11.0 (9–15.8)	0.063
Complications (≥ grade 3), n	0	4	0.104
Postoperative recurrence/metastasis, n	5	9	0.305

Data are displayed as medians with interquartile ranges, or as numbers with percentages

Propensity score matching used tumor size, stage ≥ pT2, and the presence of venous tumor thrombus as covariates

### Oncological outcomes

#### RARN vs. LRN

**Fig. 1:**
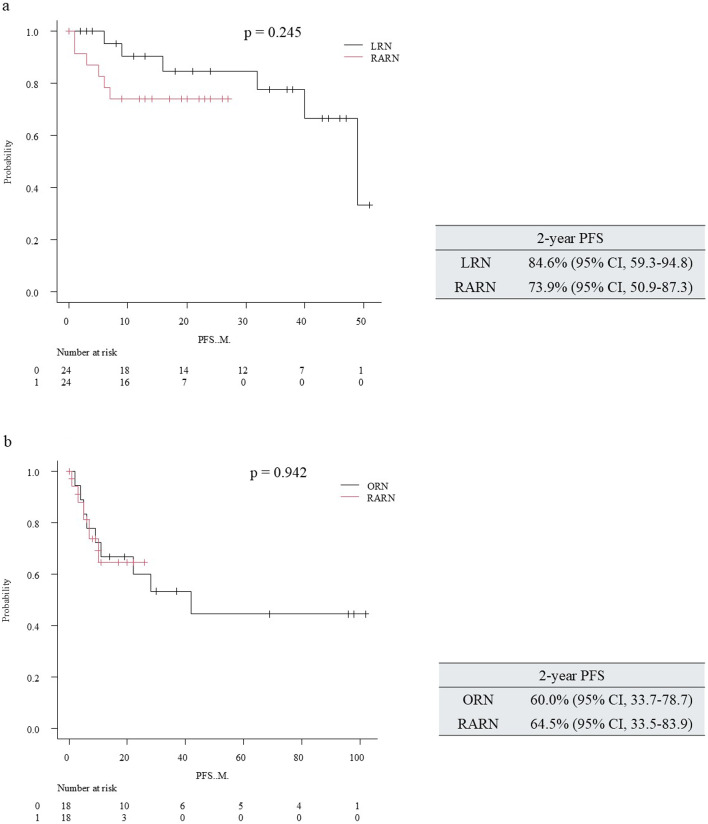
Kaplan–Meier progression-free survival after radical nephrectomy: propensity-matched comparisons of robot-assisted vs laparoscopic (**a**) and robot-assisted vs open (**b**) approaches

Primary analysis (entire period): In the PSM cohort constructed across the entire study period (covariates: tumor size, ≥pT2, venous tumor thrombi), the progression-free survival (PFS) rate was comparable between RARN and LRN (log-rank *p* = 0.245; Fig. 1a).

Sensitivity analyses: Restricting the cohort to 2023–2025 (*p* = 0.209; Supplemental Fig. 1), excluding the first five RARN cases per surgeon (*p* = 0.600; Supplemental Fig. 2), and adding metastatic status (M0/M1) to the propensity model (*p* = 0.778; Supplemental Fig. 3) all yielded consistent, nonsignificant differences.

#### RARN vs. ORN

Similarly, in the matched analysis, the two-year progression-free survival estimates were closely aligned between approaches 60.0% for the ORN cohort and 64.5% for the RARN cohort, and the log-rank comparison did not show a statistically significant difference (*p* = 0.942; Fig. 1b).

## Discussion

In this study, we compared the surgical outcomes of RARN, LRN, and ORN and found that RARN may offer advantages, particularly in complex and advanced cases. According to the National Comprehensive Cancer Network (NCCN) guidelines, RARN is regarded as a minimally invasive and fast-recovery option. However, robust evidence from randomized controlled trials (RCTs) supporting its superiority remains insufficient [9]. Similarly, the European Society for Medical Oncology (ESMO) guidelines identify RARN as a treatment option, but do not clearly define its indications [10].

Our findings align with contemporary international studies, in which robotic approaches provided perioperative advantages in appropriately selected renal cancer surgery while oncologic outcomes remained broadly comparable to conventional approaches. This is consistent with recent European series that contextualized robotic nephrectomy within real-world urologic practice [11].

Although RARN was approved for insurance coverage in Japan in 2022 and has gradually been implemented, evidence supporting optimal surgical selection between RARN, LRN, and ORN in clinical practice remains limited. A report using the U.S. National Cancer Database (NCDB) demonstrated that by 2019, RARN had become the most commonly performed radical nephrectomy approach, whereas ORN had declined significantly, suggesting that RARN may have partially replaced open surgery in selected cases [12]. A similar trend is evident at our institution, where ORN has not been performed since 2023.

According to the meta-analysis by Crocerossa and colleagues, RARN achieves cancer control similar to LRN/ORN, albeit with a tendency toward prolonged operative duration and increased costs [4]. Consistent with this, Jeong et al. documented greater expenditures for robotic nephrectomy relative to conventional approaches, highlighting potential cost-effectiveness issues [5].

Regarding the applicability of RARN in high-complexity cases, Petros et al. reported favorable outcomes in 101 patients, including those with IVC tumor thrombi and large renal masses. However, this study was conducted within a single-operator setting at a high-volume tertiary-care center; thus, caution is needed when generalizing the results [7].

In the present study, we analyzed cases of Mayo levels 0–2 IVC tumor thrombi and compared the perioperative outcomes between RARN and ORN. As shown in Supplemental Table 2, RARN resulted in significantly less blood loss (*p* < 0.001), with a trend toward shorter operative times and fewer grade ≥ 3 complications. Our findings align with earlier literature, notably those by Gu et al. and Rose et al., which demonstrated that RARN can be safely and effectively performed in selected cases with thrombi [8, 13–15]. However, cost remains a significant concern.

The interpretation of our results must consider the potential selection bias, as the RARN group included more patients with larger tumors, higher T stages, and the presence of tumor thrombi, suggesting a tendency for RARN to be selected for more advanced diseases. Our findings also indicate that robotic surgery expands the scope of surgical feasibility, even in technically challenging cases.

Therefore, RARN is not universally applicable to all nephrectomy cases. For cases amenable to LRN, especially those without an extensive tumor burden, LRN remains a cost-effective and feasible option. Conversely, RARN may represent a suitable alternative to traditional open-approach cases involving high complexity.

**Limitations.** This study had a retrospective and single-center design. Therefore, although propensity-score matching reduced baseline imbalances, residual and unmeasured confounding cannot be excluded. The matched subgroups were small, and follow-up was short with few oncologic events, so survival analyses are descriptive, and longer observation (e.g., 5-year PFS rates) is needed. Temporal and learning-curve effects may persist despite the time-restricted analysis (2023–2025) and the sensitivity analysis excluding early cases; moreover, contemporaneous ORN cases were few, which limited the precision of that comparison. Perioperative findings may also reflect institutional practice patterns, and we did not evaluate costs or surgeon-level workflow metrics, which constrains generalizability and health-economic interpretation. Prospective, multi-institutional studies with standardized protocols and comprehensive cost data are warranted to validate and extend these observations, particularly in advanced RCC.

## Conclusion

RARN appears to provide safe performance and favorable efficacy in the management of difficult or advanced renal tumors, including cases complicated by IVC tumor thrombi. Even so, because the technique entails higher expenditures and advanced operative skill, its use should be targeted rather than routine. In contrast, LRN remains appropriate and cost-effective for patients with lower-risk disease profiles. Treatment selection should be tailored on the basis of tumor burden, patient characteristics, and available institutional resources. Prospective multicenter research is essential to confirm these findings and to guide evidence-based decision-making in clinical practice.

## Supplementary Information

Below is the link to the electronic supplementary material.

Supplementary Material 1

Supplementary Material 2

Supplementary Material 3

Supplementary Material 4

Supplementary Material 5

Supplementary Material 6

Supplementary Material 7

## Data Availability

No datasets were generated or analysed during the current study.
