# Supplementary material for: Robot-assisted radical nephrectomy in comparison with open and laparoscopic approaches: a Japanese single-institution retrospective study
Source: J Robot Surg. 2025 Nov 3;19(1):745. doi: 10.1007/s11701-025-02898-x (PMC12583297; doi:10.1007/s11701-025-02898-x)
Supplement: Supplementary file 1 — Supplementary Material 1 [file 11701_2025_2898_MOESM1_ESM.docx]

**Supplemental Table 1a.** Perioperative outcomes of patients undergoing robot-assisted and laparoscopic radical nephrectomy before propensity score matching.

| Variables | RARN (n = 35) | LRN (n = 96) | p-value |
| --- | --- | --- | --- |
| Surgical approach (Transperitoneal/  Retroperitoneal) | 35 / 1 | 91 / 5 | 0.101 |
| Operative time (min), median (IQR) | 180.0 (136.5–225.5) | 151.5 (129.0–180.3) | 0.012 |
| Pneumoperitoneum time (min), median (IQR) | – | 113.5 (94–133.5) | – |
| Console time (min), median (IQR) | 100.0 (77.5–159.5) | – | – |
| Estimated blood loss (mL), median (IQR) | 58.0 (5.0–155.5) | 11.5 (4.0–40.0) | 0.018 |
| Lymph node dissection, n | 5 | 3 | 0.032 |
| Complications (≥ grade 3), n | 2 | 3 | 0.609 |
| Postoperative hospital stay (days), median (IQR) | 6.5 (5–10) | 5.0 (4–6) | <0.001 |
| Postoperative recurrence/metastasis, n | 8 | 11 | 0.158 |

Data are presented as median (interquartile range) or number.

Pneumoperitoneum and console times were not applicable in some approaches.

**Supplemental Table 1b.** Perioperative outcomes of patients undergoing robot-assisted and open radical nephrectomy before propensity score matching.

| Variables | RARN (n = 35) | ORN (n = 33) | p-value |
| --- | --- | --- | --- |
| Surgical approach (Transperitoneal / Retroperitoneal) | 35 / 1 | 32 / 1 | 0.739 |
| Operative time (min), median (IQR) | 180.0  (136.5–225.5) | 235.0  (177.0–375.0) | 0.018 |
| Console time (min), median (IQR) | 100.0 (77.5–159.5) | – | – |
| Estimated blood loss (mL), median (IQR) | 58.0 (5.0–155.5) | 1187 (752–2649) | <0.001 |
| Lymph node dissection, n | 5 | 12 | 0.050 |
| Complications (≥ grade 3), n | 2 | 6 | 0.144 |
| Postoperative hospital stay (days), median (IQR) | 6.5 (5–10) | 10 (9–16) | <0.001 |
| Postoperative recurrence/metastasis, n | 8 | 18 | 0.012 |

Data are presented as median (interquartile range) or number. Console time was not applicable for open surgery.
