# Supplementary material for: Robot-assisted radical nephrectomy in comparison with open and laparoscopic approaches: a Japanese single-institution retrospective study
Source: J Robot Surg. 2025 Nov 3;19(1):745. doi: 10.1007/s11701-025-02898-x (PMC12583297; doi:10.1007/s11701-025-02898-x)
Supplement: Supplementary file 2 — Supplementary Material 2 [file 11701_2025_2898_MOESM2_ESM.docx]

**Supplemental Table 2.** Perioperative Outcomes of RARN vs ORN in Patients with IVC Tumor Thrombus (2015–2025)

|  | RARN (n=8) | ORN (n=15) | p value |
| --- | --- | --- | --- |
| Laterality | Left: 4 Right: 4 | Left: 5 Right: 10 | 0.657 |
| Tumor thrombus Mayo level | Level 0: 5 Level I: 2 Level II: 1 | Level 0: 5 Level I: 4 Level II: 6 | – |
| Operative time (min) | 281.5 (214.3–422) | 375 (230.5–422.5) | 0.636 |
| Estimated blood loss (mL) | 209.5 (88.3–444.8) | 2556 (1515.5–2948.5) | <0.001 |
| Complications (≥ grade 3) | 0 | 3 | 0.526 |
| Postoperative hospital stay (days) | 13 (8.5–33.3) | 16 (10.5–22.5) | 0.674 |
