# Supplementary material for: Robot-assisted radical nephrectomy in comparison with open and laparoscopic approaches: a Japanese single-institution retrospective study
Source: J Robot Surg. 2025 Nov 3;19(1):745. doi: 10.1007/s11701-025-02898-x (PMC12583297; doi:10.1007/s11701-025-02898-x)
Supplement: Supplementary file 3 — Supplementary Material 3 [file 11701_2025_2898_MOESM3_ESM.docx]

Supplemental Figure 1


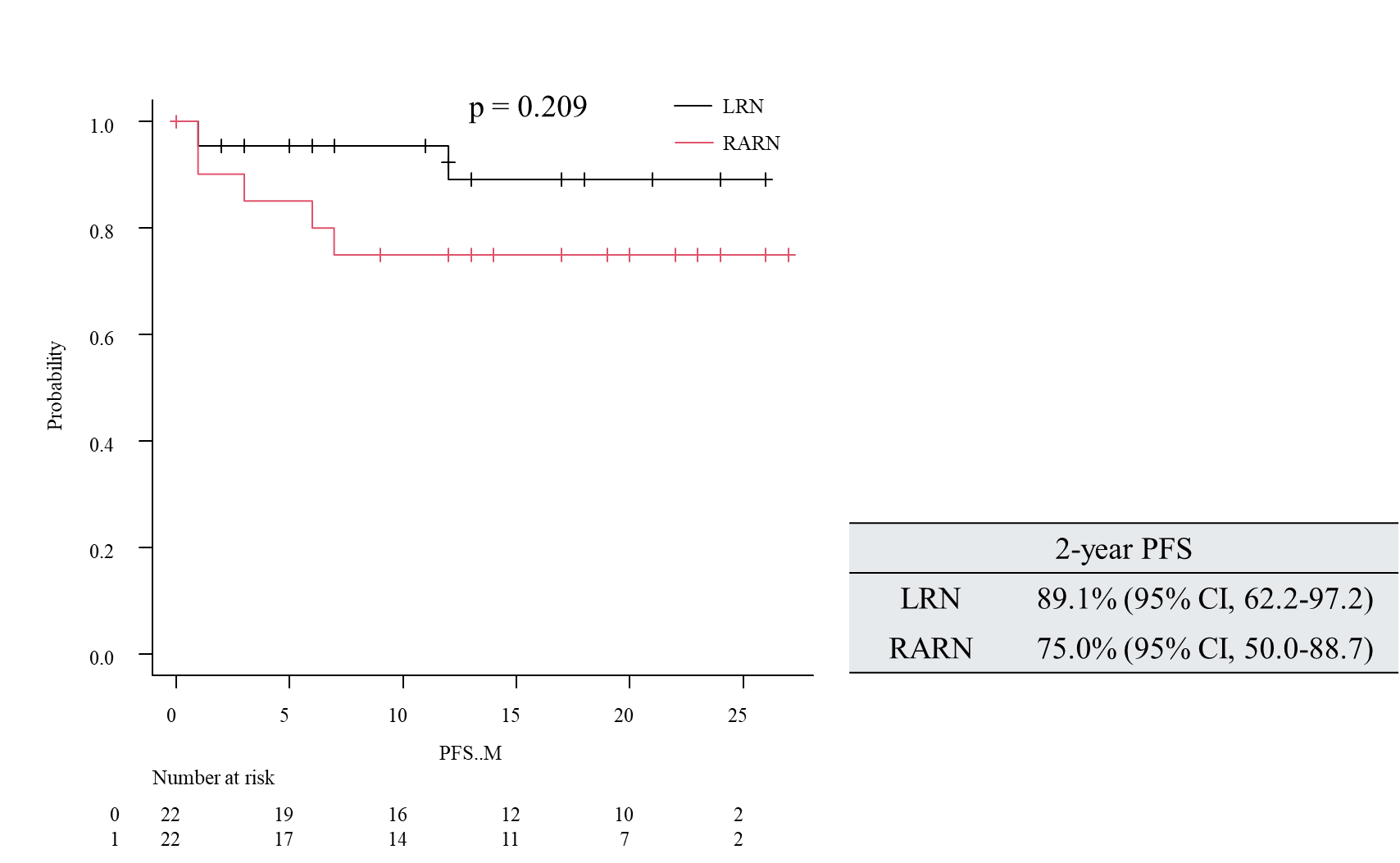


Sensitivity analysis restricted to 2023–2025: Kaplan–Meier curves for progression-free survival in the propensity-matched RARN vs LRN cohort. Matching as in the primary analysis. Log-rank p = 0.209.
