# Supplementary material for: Robot-assisted radical nephrectomy in comparison with open and laparoscopic approaches: a Japanese single-institution retrospective study
Source: J Robot Surg. 2025 Nov 3;19(1):745. doi: 10.1007/s11701-025-02898-x (PMC12583297; doi:10.1007/s11701-025-02898-x)
Supplement: Supplementary file 4 — Supplementary Material 4 [file 11701_2025_2898_MOESM4_ESM.docx]

**Supplemental Table 3a.** Baseline characteristics before propensity-score matching (time-restricted RARN vs LRN, 2023–2025).

| Factor | LRN | RARN | SMD |
| --- | --- | --- | --- |
| n | 31 | 35 |  |
| ≥ pT2 | 12 | 23 | 0.562 |
| Venous tumor thrombus | 0 | 8 | 0.770 |
| Tumor diameter (mm),  median (IQR) | 45 (22-90) | 65 (17-200) | 0.695 |

Values are n or median (IQR) as appropriate. SMD denotes the standardized mean difference; balance threshold: SMD < 0.10. These pre-matching distributions informed the matching model used in Table S3b/S3c. Abbreviations: SMD, standardized mean difference; IQR, interquartile range.

**Supplemental Table 3b.** Baseline characteristics after 1:1 propensity-score matching (time-restricted RARN vs LRN, 2023–2025).

| Factor | LRN | RARN | SMD |
| --- | --- | --- | --- |
| n | 22 | 22 |  |
| ≥ pT2 | 11 | 11 | <0.001 |
| Venous tumor thrombus | 0 | 0 | <0.001 |
| Tumor diameter (mm),  median (IQR) | 47.5 (22-90) | 50 (17-100) | 0.004 |

Matching specification: 1:1 nearest-neighbor without replacement, caliper 0.20 SD on logit (propensity score); covariates in the PS model: tumor size (continuous), ≥pT2, venous tumor thrombus. Values are n or median (IQR). All covariates met the prespecified balance criterion (SMD < 0.10). No hypothesis testing was performed on baseline variables. Abbreviations as in Supplemental Table 3a.

**Supplemental Table 3c.** Perioperative outcomes in the propensity-matched cohort (time-restricted RARN vs LRN, 2023–2025).

| Variables | RARN (n = 22) | LRN (n = 22) | p-value |
| --- | --- | --- | --- |
| Operative time (min), median (IQR) | 161 (132.3-189.3) | 158.5(146.8-168.8) | 0.639 |
| Console time (min), median (IQR) | 95 (75.3-119.3) | – | – |
| Estimated blood loss (mL), median (IQR) | 52.5 (4.3–149.5) | 4.5 (3.0–22.3) | 0.168 |
| Postoperative hospital stay (days), median (IQR) | 6.0 (4–7.8) | 4.5 (4–6) | 0.156 |
| Complications (≥ grade 3), n | 2 | 2 | 1.0 |
| Postoperative recurrence/metastasis, n | 5 | 2 | 0.412 |

Values are median (IQR) or n unless indicated. P-values reflect between-group comparisons in the matched cohort. “—” indicates not applicable (e.g., console time is specific to robotic surgery). Abbreviations: EBL, estimated blood loss; other abbreviations as in Supplemental Table 3a.
