# Supplementary material for: Robot-assisted radical nephrectomy in comparison with open and laparoscopic approaches: a Japanese single-institution retrospective study
Source: J Robot Surg. 2025 Nov 3;19(1):745. doi: 10.1007/s11701-025-02898-x (PMC12583297; doi:10.1007/s11701-025-02898-x)
Supplement: Supplementary file 5 — Supplementary Material 5 [file 11701_2025_2898_MOESM5_ESM.docx]

**Supplemental Table 4a.** Baseline characteristics before propensity-score matching (RARN vs LRN after excluding the overall first five RARN cases).

| Factor | LRN | RARN | SMD |
| --- | --- | --- | --- |
| n | 31 | 30 |  |
| ≥ pT2 | 12 | 20 | 0.583 |
| Venous tumor thrombus | 0 | 6 | 0.707 |
| Tumor diameter (mm) | 47.5 (22-90) | 67.5 (20-200) | 0.761 |

Values are n or median (IQR) as appropriate. The standardized mean difference (SMD) is shown for each covariate; balance threshold: SMD < 0.10. No hypothesis testing was performed on baseline variables.

**Supplemental Table 4b.** Baseline characteristics after 1:1 propensity-score matching (RARN vs LRN after excluding the overall first five RARN cases).

| Factor | LRN | RARN | SMD |
| --- | --- | --- | --- |
| n | 20 | 20 |  |
| ≥ pT2 | 11 | 11 | <0.001 |
| Venous tumor thrombus | 0 | 0 | <0.001 |
| Tumor diameter (mm) | 47.5 (22-90) | 49 (20-100) | 0.012 |

Matching specification: 1:1 nearest-neighbor without replacement, caliper 0.20 SD on logit (propensity score); covariates in the PS model: tumor size (continuous), ≥pT2, venous tumor thrombus. Values are n or median (IQR). All covariates met the prespecified balance criterion (SMD < 0.10). No hypothesis testing was performed on baseline variables.

**Supplemental Table 4c.** Perioperative outcomes in the propensity-matched cohort (RARN vs LRN after excluding the overall first five RARN cases).

| Variables | RARN (n = 20) | LRN (n = 20) | p-value |
| --- | --- | --- | --- |
| Operative time (min), median (IQR) | 161 (131.5-192.5) | 158.5(135.3-168.3) | 0.561 |
| Console time (min), median (IQR) | 91 (74.8-106.3) | – | – |
| Estimated blood loss (mL), median (IQR) | 52.5 (2-142.5) | 6 (3-28.5) | 0.471 |
| Postoperative hospital stay (days), median (IQR) | 5.5 (4-7.3) | 4.5 (4–5.3) | 0.171 |
| Complications (≥ grade 3), n | 2 | 1 | 1.0 |
| Postoperative recurrence/metastasis, n | 3 | 2 | 1.0 |

Values are median (IQR) or n, as indicated. P-values reflect between-group comparisons in the matched cohort. “—” indicates not applicable (e.g., console time is specific to robotic surgery and not recorded for LRN).
