# Supplementary material for: Robot-assisted radical nephrectomy in comparison with open and laparoscopic approaches: a Japanese single-institution retrospective study
Source: J Robot Surg. 2025 Nov 3;19(1):745. doi: 10.1007/s11701-025-02898-x (PMC12583297; doi:10.1007/s11701-025-02898-x)
Supplement: Supplementary file 6 — Supplementary Material 6 [file 11701_2025_2898_MOESM6_ESM.docx]

Supplemental Figure 3


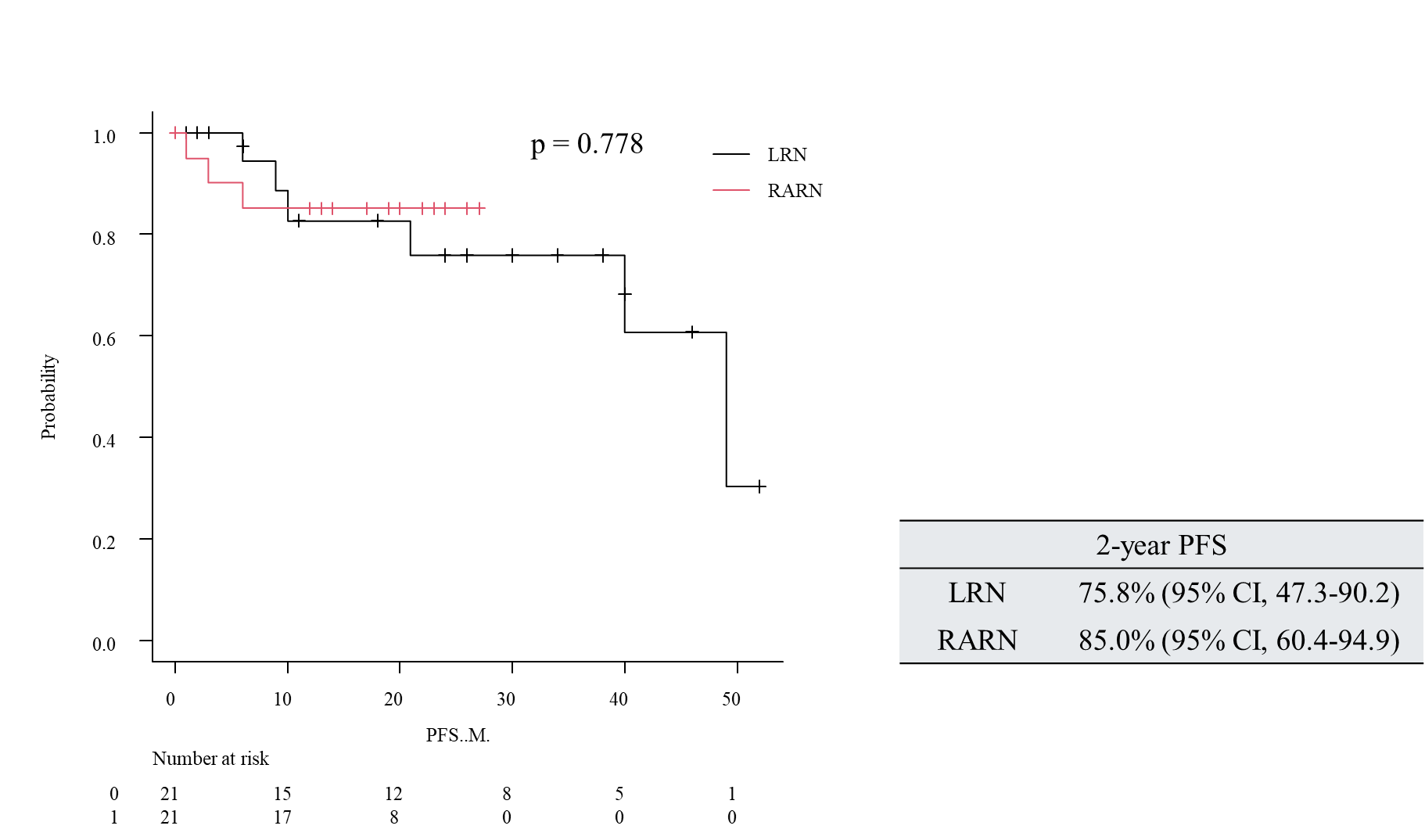


Sensitivity analysis adding metastatic status (M0/M1) to the propensity model (no time restriction): Kaplan–Meier curves for progression-free survival. Log-rank p = 0.778. Two-year PFS: LRN 75.8% (95% CI 47.3–90.2) vs RARN 85.0% (60.4–94.9).
